# Comparative Assessment of DNA Targets and Amplification Methods for *Leishmania (Viannia)* Detection in Human Samples

**DOI:** 10.4269/ajtmh.19-0691

**Published:** 2020-03-30

**Authors:** Mariana Rosales-Chilama, Nicole Diaz-Moreno, Miguel Darío Prieto, Lina Giraldo-Parra, Álvaro José Martínez-Valencia, María Adelaida Gomez

**Affiliations:** 1 Centro Internacional de Entrenamiento e Investigaciones Médicas-CIDEIM, Cali, Colombia;; 2 Universidad Icesi, Cali, Colombia

## Abstract

Multiple polymerase chain reaction (PCR)-based approaches have been developed for *Leishmania* detection in clinical and laboratory samples, and this diversity limits inter-study comparisons, meta-analyses, and generalization of findings. Towards harmonization of a molecular tool for detection of *Leishmania (Viannia)* for research purposes*,* we evaluated the concordance of 18SrDNA quantitative polymerase chain reaction (qPCR) and minicircle kinetoplastid DNA (mkDNA) PCR followed by Southern blot (PCR-SB) in in vitro infection systems and in lesion and mucosal swab samples from Colombian patients with cutaneous leishmaniasis caused by *L. (Viannia)*. The lower limit of parasite detection of 18SrDNA qPCR and mkDNA PCR-SB was 10^−1^ promastigotes and one intracellular amastigote per reaction. From cutaneous lesions (*n* = 63), an almost perfect concordance was found between the methods (κ = 0.92, 95% CI: 0.82–1.00). Despite equal limits of detection, mkDNA PCR-SB was more efficient for parasite detection in mucosal samples than 18SrDNA qPCR or 18SrDNA digital droplet PCR. The high concordance, sensitivity, scaling potential, and feasibility of implementation of the 18SrDNA qPCR, support its selection as the *L.* (*Viannia*) in research laboratories, as a first step towards harmonization of research protocols in the region.

## INTRODUCTION

PCR-based tools for detection of *Leishmania* in clinical and experimental samples are more sensitive, less labor intensive, and amenable for medium- to high-throughput scaling compared with the standard microscopic examination of tissues or infected cells in glass slides. Therefore, molecular parasite detection has been widely used as the preferred readout for drug susceptibility screening assays, host–pathogen interaction outcomes, pathogenicity and virulence studies, and assessment of parasite persistence, among many others.^1–4^

The array of reported and used molecular targets for *Leishmania* detection (kDNA, 18SrDNA, 7SLRNA, internal transcribed spacer [ITS], mini-exon, HSP70, GP63, and others)^1^ and the number of amplification methods and parameters used in clinical and research laboratories (including definition of the amplification approach, selection of the visualization method, and definition of the reporting system and scale) limit the objective comparison, interpretation, and meta-analysis of studies and results. Unlike the standardized procedures developed and applied in clinical microbiology such as international protocols for minimal inhibitory concentration testing in bacteria,^5^ the diversity of *Leishmania* detection tools restricts harmonization of methods for defining clinically relevant parameters such as drug susceptibility break points, relationships between parasite load and virulence, or the magnitude and impact of parasite persistence.

Among the most commonly used molecular targets for *Leishmania* detection are the 18SrDNA and minicircle kinetoplastid DNA (mkDNA). Because of the high copy number (∼10,000 copies/parasite cell), detection of mkDNA has been the most widely used molecular surrogate of the presence of *Leishmania*.^4,6,7^ However, its usefulness as a screening target is compromised because of sequence homology with other kinetoplastids,^6^ high background amplifications, and reported cross-reactivity of some primers with human DNA.^6,8^ To overcome this barrier, technically complex procedures, such as Southern blot, and the use of specialized infrastructure and software-like high-resolution melting have been developed; however, they have limited potential of implementation and dissemination because of technology access barriers.^9^

A less complex and laborious method for *Leishmania* detection is qPCR amplification of the 18SrDNA, which has been successfully tested for the diagnosis of American cutaneous leishmaniasis (ACL), with sensitivity and specificity of 98% and 84%, respectively, compared with the gold standard of parasite detection in lesion smears and/or parasite isolation.^10^ However, its performance alongside the apparently more sensitive amplification of mkDNA coupled to Southern blot (PCR-SB) has not been systematically evaluated. In this study, we sought to determine the concordance of *Leishmania (Viannia)* detection by qPCR amplification of 18SrDNA and mkDNA PCR-SB in samples from patients with suspected ACL from endemic areas of *L. (Viannia)* transmission in Colombia, aiming to provide a standardized tool for clinical specimens and culture-derived samples, toward harmonization of a molecular tool for *L. (Viannia)* detection for research purposes*.*

## MATERIALS AND METHODS

### Study participants and samples.

This study included banked DNA samples collected from a total of 63 participants, men, or women with parasitological diagnosis of ACL (*n* = 47) and with ulcerated skin lesions of other etiology (non-ACL, *n* = 16) of any age and any ethnic background (Table 1). Two swab samples of one ulcerated lesion were taken from all ACL and non-ACL patients, and in 29 ACL patients, duplicate swab samples from tonsil and nasal mucosal tissues were also obtained. All patients diagnosed with ACL were treated according to current Colombian guidelines. A post hoc power analysis of our sample (*n* = 63) based on the 95% CI was performed to measure a kappa coefficient of 0.8 between tests, assuming a probability of 0.75 positive cases (“kappaSize” R package, v. 1.2). As calculated, the lower limit of a 95% CI was 0.612, providing confidence in finding a substantial (0.61–0.8) or almost perfect agreement (0.81–1) in the concordance analysis.

**Table 1:** Clinical and demographic characteristics of study participants

Characteristic	Total	ACL	Non-ACL	*P*-value
Number of subjects, *n* (%)	63	47 (74.6)	16 (25.4)	
Gender, *n* (%)				
Male	51 (81.0)	41 (87.2)	10 (62.5)	0.059*
Female	12 (19.0)	6 (12.8)	6 (37.5)	
Age in years, median (range)	26 (3–70)	26 (3–57)	25.5 (5–70)	0.538†
Ethnicity, *n* (%)				
Afro-Colombian	28 (44.4)	21 (44.7)	7 (43.8)	
Mestizo	28 (44.4)	20 (42.6)	8 (50.0)	0.999*
Indigenous	6 (9.5)	5 (10.6)	1 (6.2)	
Caucasian	1(1.6)	1 (2.1)	0	
Time from onset of lesions in months, median (range)	2 (0.2–240)	1 (0.5–12)	3 (0.2–240)	0.063†
Number of lesions, median (IQR)	1 (1–12)	1 (1–6)	2 (1–12)	0.566†
CL Diagnostic method, *n* (%)				
Positive culture and smear	NA	33 (70.2)	NA	
Positive smear only		9 (19.2)		
Positive culture only		5 (10.6)		
*Leishmania* strain isolated				
*Leishmania (Viannia) panamensis*	NA	31 (81.6)	NA	
*Leishmania (Viannia) braziliensis*		5 (13.2)		
Unavailable		2 (5.2)		

ACL = American cutaneous leishmaniasis; IQR = interquartile range.

* Chi-squared test/Fisher’s test.

† *t*-test/Wilcoxon rank sum test.

### *Leishmania* strains.

Seven *Leishmania* and one *Trypanosoma* reference strains were obtained from CIDEIM BioBank: *Leishmania (Viannia) panamensis* (MHOM/PA/71/LS94), *Leishmania (Viannia) guyanensis* (MHOM/BR/75/M4147), *Leishmania (Viannia) braziliensis* (MHOM/BR/75/M2903), *Leishmania (Leishmania) infantum* (MHOM/BR/74/PP75), *Leishmania (Leishmania) amazonensis* (MHOM/BR/73/M2269), *Leishmania (Leishmania) mexicana* (MHOM/BZ/82/BEL21), *Leishmania (Leishmania) donovani* (MHOM/IN/80/DD8), and *Trypanosoma cruzi* (MHOM/CH/00/Tulahuen). Promastigotes and trypomastigotes were maintained at 25°C in complete Roswell Park Memorial Institute (RPMI) medium (supplemented with 10% heat-inactivated fetal bovine serum [Gibco], 1% glutamine, 100 U/mL penicillin, and 100 μg/mL streptomycin). Logarithmic phase promastigotes and trypomastigotes were harvested by centrifugation, washed in PBS, and solubilized in lysis buffer for DNA extraction.

### THP-1 culture and infection.

Human monocytic cell line derived from an acute monocytuc leukemia patient (THP-1) cells were maintained at 1 × 10^6^ cells/mL in complete RPMI at 37°C and 5% CO_2_ and infected with human AB+ serum-opsonized stationary phase *L. (V.) panamensis* (MHOM/COL/03/3594/LUC001) at a 10:1 *Leishmania*-THP-1 ratio for 24 hours at 34°C with 5% CO_2_.

### DNA extractions, amplification, and detection.

DNeasy Blood & Tissue Kit and AllPrep DNA/RNA Mini Kit (Qiagen, Hilden, Germany) were used for extractions. Standard curves of 18SrDNA and mkDNA were constructed by 2-fold serial dilution of DNA products obtained from *L. (V.) panamensis* promastigotes (L.p.-Luc001) and infected THP-1 cells. Three to five independent replicas of these standard curves were generated from independent culture preparations to assess reproducibility of the dynamic range of each method. Minicircle kinetoplastid DNA was amplified by PCR using the LV-B1 primers, followed by Southern blot hybridization as follows: each 25 μL of the PCR reaction mixture contained 0.8 mM of dNTP, 0.04 U/μL Taq DNA polymerase (Invitrogen), 2 μL template DNA, 2 mM MgCl_2_, 1× PCR buffer, and 0.4 nM of LV and B1 primers (primer sequences: LV 5′-ATTTTTGAACGGGGTTTCTG-3′ and B1 5′-GGGGTTGGTGTAATATAGTGG-3′). The cycling reaction was as follows: 95°C for 5 minutes, followed by 35 cycles each of 1 minute at 92°C, 40 seconds at 60.5°C and 30 seconds at 72°C, and a final extension of 1 minute at 72°C. Southern blotting of PCR products was performed using standard procedures using an mkDNA detection probe derived from *L. (V.) panamensis*.^8^ *Leishmania* 18SrDNA was amplified by qPCR as follows: reactions were conducted in a total volume of 12.5 μL, containing 1.25 μL of the DNA sample, 6.25 μL PCR Mastermix (BioRad, Irvine, CA), 0.8 μM of each of the two primers designed to amplify *Leishmania* 18SrDNA and 0.2 μM of the *Leishmania* 18SrDNA-specific FAM-labeled TaqMan probe (Frw primer: 5′CCAAAGTGTGGAGATCGAAG3′, Rev primer: 5′GGCCGGTAAAGGCCGAATAG3′, probe: 5′6-FAM-ACCATTGTAGTCCACACTGC-NFQ-MGB 3′). qPCR was performed on a BioRad CFX96 platform as follows: denaturation at 95°C for 10 minutes, followed by 35 cycles of 95°C for 15 seconds, and 60°C for 50 seconds, including FAM detection.^10^ Digital droplet PCR (ddPCR) was performed using the Bio-Rad QX100 workflow.^11^ The same DNA aliquot was evaluated by the different molecular methods. DNA carryover/contamination was controlled by inclusion of blank (water) and negative controls (DNA of THP-1 cells). Human glyceraldehyde-3-phosphate dehydrogenase (GAPDH) was amplified from mucosal samples as quality control of samples with suspected low parasite burden.^4^

### Statistical analyses.

Differences in frequencies were evaluated using Fisher’s exact tests and *t*-test/Wilcoxon rank sum tests for unpaired data. Concordance was estimated by the kappa coefficient test and 95% CIs (R software v. 3.4.3 and base packages). Interpretation of kappa values was done as previously described^12^: values ≤ 0 indicating no agreement, 0.01–0.20 none to slight, 0.21–0.40 fair, 0.41–0.60 moderate, 0.61–0.80 substantial, and 0.81–1.00 almost perfect agreement.

## RESULTS AND DISCUSSION

**Figure 1.**
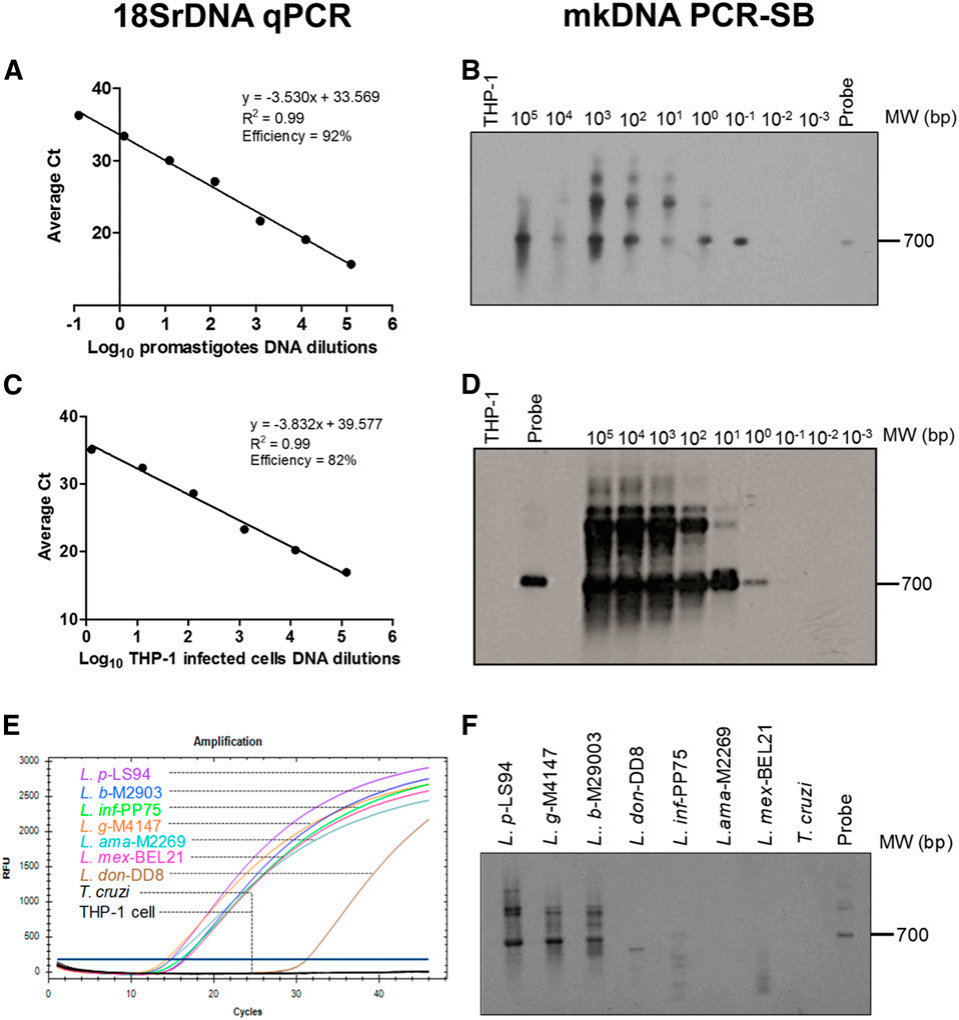
Limit of detection and specificity of 18SrDNA qPCR and minicircle kinetoplastid DNA (mkDNA) PCR followed by Southern blot (PCR-SB). The detection limits of 18SrDNA qPCR (**A** and **C**) and mkDNA PCR-SB (**B** and **D**) were quantified using 2-fold serial dilution of DNA products obtained from (**A** and **B**) 1 × 10^7^ L.p.-Luc001 promastigotes and (**C** and **D**) L. p.-Luc001–infected THP-1 cells. Representative images of at least three independent standard curves are shown. Specificity of (**E**) 18SrDNA and (**F**) mkDNA amplification was evaluated with a reference strain panel consisting of: *Leishmania (Viannia) panamensis (L. p)*, *Leishmania (Viannia) guyanensis (L. g)*, *Leishmania (Viannia) braziliensis (L. b)*, *Leishmania (Leishmania) donovani (L. don)*, *Leishmania (Leishmania) infantum (L. inf)*, *Leishmania (Leishmania) amazonensis (L. ama)*, *Leishmania (Leishmania) mexicana (L. mex)*, and *Trypanosoma cruzi (T. cruzi).* A 700-bp band corresponds to the full-length amplification product of *Leishmania* mkDNA in the PCR-SB images; additional bands correspond to unspecific mkDNA PCR products. This figure appears in color at www.ajtmh.org.

The detection limits of 18SrDNA qPCR and mkDNA PCR-SB were determined using DNA extracts from axenically cultured *L. (V.) panamensis* promastigotes and infected THP-1 cells. Both methods detected up to 10^−1^ promastigotes (Figure 1A and B) and one intracellular amastigote per reaction, respectively (Figure 1C and D). Because of the presence of multiple copies of the mkDNA and 18SrDNA targets in each parasite cell, detection limits can be less than one parasite per reaction. The primers used for both 18SrDNA and mkDNA amplification did not cross-react with human DNA (THP-1 cells) (Figure 1B–D). As previously shown, amplification of the 18SrDNA target allowed detection of species of both *L. (Leishmania)* and *L. (Viannia)* subgenus and did not cross-react with *T. cruzi* DNA (Figure 1E). Conversely, the PCR-SB of mkDNA was specific only for species of the *Viannia* subgenus, visualized by amplification of a 700-bp product in *L. (V.) panamensis*, *L. (V.) braziliensis*, and *L. (V.) guyanensis.* Nonspecific amplification products of lower molecular weight were detected in *L. (L.) donovani*, *L. (L.) infantum*, and *L. (L.) mexicana* samples (Figure 1F).

Lesion swab samples from 47 patients from endemic regions of *L. (Viannia)* transmission in Colombia, with parasitological diagnosis of ACL, and 16 with other ulcerated cutaneous pathologies (Table 1) were analyzed. Of samples from ACL patients, 45 were equally classified by 18SrDNA qPCR and mkDNA PCR-SB: 42 in which *Leishmania* was detected by both methods and three where parasites were not detected, despite parasitological diagnosis by lesion smear. Two samples were not concordant, both being positive by mkDNA PCR-SB and negative by 18SrDNA. Of the 16 samples from non-ACL patients, 14 samples were *Leishmania* negative by 18SrDNA qPCR and mkDNA PCR-SB, whereas two were positive by both molecular methods (despite being *Leishmania* negative by tissue smear or culture of lesion aspirate). Together, the concordance of the two molecular tests in all samples (*n* = 63) was almost perfect (κ = 0.92).

The presence of *Leishmania* in nasal mucosa, tonsils, and conjunctiva of ACL patients typically presents with low parasite burden ranging from 0.2 to 22 parasites/reaction,^4^ and thus, mkDNA PCR-SB has been the primary screening tool used.^13^ We explored whether 18SrDNA qPCR was equally concordant to mkDNA PCR-SB in these sample types. Swab samples from nasal mucosa and tonsils from 29 ACL patients were evaluated. Amplification of human GAPDH gene was positive in 93% of samples (54 of 58), corroborating good quality of the extracted DNA. GAPDH-negative samples were excluded from further analysis. Two samples (2 of 54; 7%), one tonsil and one nasal sample, each from a different patient, were positive by mkDNA PCR-SB. Only the nasal mucosa sample was positive by 18SrDNA qPCR. Considering the higher sensitivity of ddPCR versus qPCR, the two non-concordant and the three negative samples, together with six randomly selected qPCR- and PCR-SB–positive samples, were also analyzed by 18SrDNA ddPCR.^14^ All samples were equally classified by 18SrDNA qPCR and ddPCR (six positive and five negative samples), and two remained non-concordant against mkDNA PCR-SB (both positive by mkDNA PCR-SB and negative by 18SrDNA qPCR and ddPCR). No differences were found between qPCR and ddPCR 18SrDNA amplification, consistent with recent findings.^11^ Because of the limited sample size, concordance analysis was not possible and further sampling and testing is encouraged.

**Table 2:** Concordance of 18SrDNA and kDNA for detection of *Leishmania* from lesion swab samples

Sample	18SrDNA qPCR	kDNA PCR-SB		Total	Kappa (95% CI)
		Positive	Negative		
Confirmed ACL	Positive	42	0	47	0.73 (0.37–1.00)
	Negative	2	3		
Non-ACL	Positive	2	0	16	1.00 (1.00–1.00)
	Negative	0	14		
All samples	Positive	44	0	63	0.92 (0.82–1.00)
	Negative	2	17		

ACL = American cutaneous leishmaniasis; CI = confidence interval.

Results from this study show an almost perfect concordance between mkDNA PCR-SB and 18SrDNA qPCR for detecting *Leishmania* in lesion samples from CL patients caused by *L. (V.) panamensis* and *L. (V.) braziliensis* in Colombia and equal detection limits in in vitro cell cultures. Beyond the clinical relevance, quantitative and qualitative molecular detection of *Leishmania* constitute central tools for exploring, among others, the natural history of infection (including symptomatic and asymptomatic infections), the epidemiology of transmission, and antileishmanial drug activity and its effects. Therefore, the harmonization of molecular methods and tools for parasite detection is essential for translational research activities that require interlaboratory comparisons, meta-analysis, or the definition and implementation of clinically relevant break points and methods. Our results support the use of 18SrDNA qPCR over mkDNA-SB for detection of *L. (V.) panamensis* and *L. (V.) braziliensis* in samples with expected parasite burden > 1 parasite/reaction, such as lesion samples, in vitro cell cultures, and experimental infections.

The amplification of RNA versus DNA targets for the detection of microbes in clinical specimens and experimental samples has been a matter of discussion and controversy.^15,16^ It is suggested that the detection of *Leishmania* DNA molecules may introduce false-positive results because of the stability of DNA molecules beyond cell death.^15,16^ Despite this, the higher stability of DNA favors sensitivity for detection of parasite gene targets. By contrast, the short half-life and lability of RNA molecules allow the association between cell viability and RNA detection to be established. As a consequence, the instability of RNA transcripts limits the feasibility of parasite detection in substandard RNA preparations, such as lesion biopsy samples.^15,16^ Therefore, we propose that DNA detection methods, such as those presented in this study, be used as screening tools for *Leishmania* detection and estimation of overall parasite loads. If required, *Leishmania* detection can be complemented with subsequent amplification of RNA molecules for the validation of viability and accurate quantification of absolute parasite numbers.^4,6^
